# Leptospirosis in Latin America: exploring the first set of regional data

**DOI:** 10.26633/RPSP.2017.81

**Published:** 2017-06-19

**Authors:** Maria Cristina Schneider, Deise Galan Leonel, Patricia Najera Hamrick, Eduardo Pacheco de Caldas, Reina Teresa Velásquez, Fernando Antonio Mendigaña Paez, Jusayma Caridad González Arrebato, Andrea Gerger, Martha Maria Pereira, Sylvain Aldighieri

**Affiliations:** 1 Pan American Health Organization Department of Communicable Diseases and Health Analysis Washington, D.C. United States of America Pan American Health Organization, Department of Communicable Diseases and Health Analysis, Washington, D.C., United States of America; 2 Ministério da Saúde Secretaria de Vigilância em Saúde Brasília Brasil Ministério da Saúde, Secretaria de Vigilância em Saúde, Brasília, Brasil.; 3 Secretaría de Salud Tegucigalpa Honduras Secretaría de Salud, Tegucigalpa, Honduras; 4 Ministerio de Salud y Protección Social Subdirección de Enfermedades Transmisibles Bogotá Colombia Ministerio de Salud y Protección Social, Subdirección de Enfermedades Transmisibles, Bogotá, Colombia; 5 Ministerio de Salud Pública Programa Nacional de Zoonosis La Habana Cuba Ministerio de Salud Pública, Programa Nacional de Zoonosis, La Habana, Cuba; 6 Fundação Oswaldo Cruz Centro Colaborador da Organização Mundial da Saúde para Leptospirose Rio de Janeiro Brasil Fundação Oswaldo Cruz, Centro Colaborador da Organização Mundial da Saúde para Leptospirose, Rio de Janeiro, Brasil.

**Keywords:** Leptospirosis, zoonoses, government programs, health surveillance system, Latin America, Leptospirosis, zoonosis, programas de gobierno, sistema de vigilancia sanitaria, América Latin, Leptospirose, zoonoses, programas governamentais, sistema de vigilância sanitária, América Latina

## Abstract

**Objectives.:**

*To demonstrate the importance of country surveillance systems for leptospirosis and their use for preliminary epidemiological analysis, as well as to generate research questions for future, morecomprehensive studies on the disease*.

**Methods.:**

*In 2015, for the first time, the Pan American Health Organization (PAHO) included human cases of leptospirosis in its Regional Core Health Data Initiative, an open-access database that collects annual health indicators from the countries and territories of the Americas. This new information was used to analyze leptospirosis cases by country and sex and to calculate cumulative incidence rates. Maps were used to help present the results. To supplement that general review of leptospirosis in the Americas, more detailed descriptions of the epidemiological situation and the surveillance programs of four selected countries (Brazil, Colombia, Cuba, and Honduras) were provided*.

**Results.:**

*In this first year of PAHO requesting leptospirosis data, of the 49 countries and territories in the Americas, 38 of them (77.6%) reported information. Among those 38, 28 of them (73.7%) reported the presence of human cases; the majority of instances of zero cases were in Caribbean territories. From those 28, a total of 10 702 human cases were recorded. The largest numbers of cases in Latin America were in Brazil (40.2%), Peru (23.6%), Colombia (8.8%), and Ecuador (7.2%). The cumulative incidence rate for Latin America was estimated to be 2.0 per 100 000 population. On average, 65.1% of cases were males*.

**Conclusions.:**

*This study demonstrates that many countries in Latin America are making efforts to establish strong surveillance systems and programs for leptospirosis. The study also shows the importance of having leptospirosis surveillance systems as well as how the information generated can be used for evidence-based decision-making on leptospirosis*.

## RESUMEN

***Objetivos*.:**

Demostrar la importancia de los sistemas nacionales de vigilancia de la leptospirosis y su uso para realizar el análisis epidemiológico preliminar, así como para generar preguntas de investigación que se utilicen en futuros estudios más integrales sobre la enfermedad.

***Métodos*.:**

En el año 2015, la Organización Panamericana de la Salud (OPS) incluyópor primera vez casos humanos de leptospirosis en su Iniciativa Regional de Datos Básicos de Salud, base de datos de acceso abierto que recoge indicadores de salud anuales de los países y territorios de la Región de las Américas. Esta nueva información fue utilizada para analizar los casos de leptospirosis por país y sexo, así como para calcular las tasas de incidencia acumulada, y los resultados se presentaron en mapas. Para complementar ese examen general de la leptospirosis en esta Región, se aportó una descripción más detallada de la situación epidemiológica y los programas de vigilancia de cuatro países (Brasil, Colombia, Cuba y Honduras).

En este primer año en que la OPS solicitó datos sobre la leptospirosis, de los 49 países y territorios en la Región de las Américas, aportaron información 38 (77,6 %). De esos 38, 28 (73,7 %) notificaron casos humanos; la mayor parte de las instancias sin ningún caso humano se registraron en territorios del Caribe. En esos 28 países y territorios, se registraron 10 702 casos humanos. En América Latina, los países que registraron los números más altos de casos fueron Brasil (40,2 %), Perú (23,6 %), Colombia (8,8 %) y Ecuador (7,2 %). Se calculó que la tasa de incidencia acumulada de América Latina es de 2,0 por 100 000 habitantes. En promedio, 65,1 % de los casos correspondieron a hombres.

***Conclusiones*.:**

Este estudio demuestra que muchos países de América Latina están tomando medidas para instaurar sistemas y programas sólidos de vigilancia de la leptospirosis. Asimismo, revela la importancia de los sistemas de vigilancia de la leptospirosis, así como el modo en que la información generada puede servir para tomar decisiones basadas en la evidencia acerca de esta enfermedad.

## RESUMO

**Objetivos.:**

Demonstrar a importância dos sistemas nacionais de vigilância para a leptospirose e seu uso na análise epidemiológica preliminar, além de gerar perguntas de pesquisa para futuros estudos mais aprofundados sobre a doença.

**Métodos.:**

Em 2015, a Organização Pan-Americana da Saúde (OPAS) incluiu, pela primeira vez, casos humanos de leptospirose em sua Iniciativa Regional de Dados Básicos de Saúde, uma base de dados de acesso aberto que coleta indicadores de saúde anuais nos países e territórios das Américas. Utilizamos estas novas informações para analisar os casos de leptospirose por país e por sexo e calcular as taxas de incidência acumuladas. A apresentação dos dados é facilitada pelo uso de mapas. Para complementar esta revisão geral da leptospirose nas Américas, apresentamos descrições mais detalhadas da situação epidemiológica e dos programas de vigilância de quatro países selecionados (Brasil, Colômbia, Cuba e Honduras).

**Resultados.:**

Neste primeiro ano em que a OPAS solicitou dados sobre a leptospirose, 38 dos 49 países e territórios das Américas (77,6%) apresentaram informações. Destes 38, 28 (73,7%) notificaram a presença de casos humanos; em sua maioria, os que não apresentaram nenhum caso foram territórios do Caribe. Dentre os 28, foi registrado um total de 10.702 casos humanos. O maior número de casos na América Latina foi observado no Brasil (40,2%), Peru (23,6%), Colômbia (8,8%) e Equador (7,2%). A taxa de incidência acumulada para a América Latina foi estimada em 2,0 por 100.000 habitantes. Em média, 65,1% dos casos foram em homens.

**Conclusões.:**

Este estudo demonstra que muitos países da América Latina estão fazendo esforços para estabelecer fortes sistemas de vigilância e programas contra a leptospirose. O estudo também revela a importância da existência de sistemas de vigilância para a leptospirose e demonstra que as informações geradas podem ser usadas para a tomada de decisões baseadas em evidências científicas para a leptospirose.

In order to support decisionmakers in prioritizing infectious diseases for allocating resources and encouraging interventions at different levels, it is necessary to make the diseases discernible. Leptospirosis is one of the most common zoonotic infectious diseases worldwide, usually transmitted through animal urine in the environment. However, at the same time, it is a neglected disease, since researchers still lack a large amount of information, and country surveillance systems are not always in place.

A recently published systematic review concluded that leptospirosis is one of the leading zoonotic causes of morbidity and mortality around the world, and estimated that the greatest burdens are in resource-poor regions and in areas where surveillance is not routinely performed (1). Overall, leptospirosis was estimated to cause approximately 1.0 million cases and 58 900 deaths each year worldwide. In the Americas, the annual estimated morbidity rates ranged from 3.9 per 100 000 population in southern Latin America to 50.7 per 100 000 population in the Caribbean (1).

In Latin America, leptospirosis may be categorized into one of two major profiles, both poverty related. The first scenario, usually more noticeable to health authorities and the media, involves cases that occur after heavy rains, floods, or other natural disasters, which frequently affect urban areas with poor infrastructures (2–5). The second scenario refers to occupationally acquired leptospirosis, for example in paddy field workers, which is typically not covered by the media and occurs more commonly in resource-deficient rural areas (6, 7).

Leptospirosis is an important disease in Latin America, given its epidemiology, with an association with tropical and subtropical ecosystems and links to such risk factors as floods and urine-polluted environments (8). Annually, 10 million people are affected by natural disasters in the Region of the Americas. Of those disasters, 41.0% are storms and 35.0% are floods (9). In addition, Latin America is one of the larger rice producers in the world (10), and the majority of its territory is covered by tropical and subtropical terrestrial ecoregions (11).

A review conducted using HealthMap, a global database that utilizes different online sources for real-time surveillance of emerging public health threats (http://www.healthmap.org/en/), concluded that, for the period of 2007 to 2013, 63.0% of the global alerts reported on leptospirosis were for the Americas (12).However, accurate data on leptospirosis is very limited in the Americas (13). In 2015, for the first time, the Pan American Health Organization (PAHO) included human leptospirosis cases in its Regional Core Health Data Initiative (14). Focused on the health situation and trends in the countries of the Americas, that information platform is a joint collective effort of PAHO and its Member States. This is the first time that, for Latin America, a set of leptospirosis cases based on country surveillance data has been presented.

The objectives of this study were to demonstrate the importance of country surveillance systems for leptospirosis and their use for preliminary epidemiological analysis in the Americas, as well as to generate research questions for future, more comprehensive studies.

## MATERIALS AND METHODOS

### Criteria

In 2015, the PAHO Regional Core Health Data Initiative included leptospirosis cases in its annual request for official information from the 49 countries and territories of the Region of the Americas (14).

The number of leptospirosis cases reported was according to the respective country surveillance systems. Diagnosis is usually based on clinical signs and symptoms, in conjunction with laboratory confirmation and epidemiological data (a history of possible exposure and presence of risk factors). The “gold standard” microscopic agglutination test (MAT) and the enzyme-linked immunosorbent assay (ELISA) are two serologic tests recommended by the World Health Organization (WHO) for laboratory diagnosis of leptospirosis (8).

Prior to 2015, the leptospirosis information available for the Region of the Americas was from the alerts from HealthMap (15). We have included in our analysis a consolidation of the alerts from 2010 to 2014, by country. In order to identify the Latin American countries with the highest risk for leptospirosis, we authors developed a set of groupings basedon the available information. High-risk countries were characterized as being in the highest quartile for one or more of the following criteria:a) number of cases in 2014, b) cumulative incidence rates in 2014, and c) number of HealthMap alerts for 2010 to 2014.

To exemplify some of the Latin American countries considered as high risk for leptospirosis according the above criteria, the government program structure and disaggregated epidemiological situation are summarized later in this piece in profiles of four countries: Brazil, Colombia, Cuba, and Honduras.

### Data analysis

The PAHO Regional Core Health Data Initiative divides the 49 countries and territories of the Americas into three groups: North America (3 countries/territories); Latin America (including the Latin Caribbean) (25 countries/territories); and the non-Latin Caribbean (21 countries/territories) (16). Below, we present a general overview of the data received from all the countries reporting data. However, this study is mainly focused on 21 Latin American countries, with 4 small-population territories of the Latin Caribbean not included in our analysis. The country of Belize was also excluded since it did not report official data on leptospirosis for 2014. Therefore, this article reports on the leptospirosis epidemiological situation from a total of 20 countries in Latin America.

Absolute numbers and estimated cumulative incidence rates per 100 000 population are presented by country. Leptospirosis cases were analyzed by sex and by country for the countries with 10 or more cases reported. Selected socioeconomic indicators were included to illustrate the analysis and to generate questions for future observational studies. Analyses were conducted using Microsoft Excel software. Maps comparing number of cases, incidence rates, and number of alerts from HealthMap were produced over a terrestrial ecoregions background, using ArcGIS 10.4 software.

### Limitations

One of the limitations of this study is that not all countries have strong surveillance systems for this disease. In addition, those systems may differ among and within countries. For example, despite the fact that the WHO recommends the gold standard MAT, laboratory facilities and diagnostic techniques can be different even for diagnostic laboratories at the same subnational level within a country.

## RESULTS

In 2015, the first year that the PAHO Regional Core Health Data Initiative requested information on the number of leptospirosis cases in the Region of the Americas, 38 out of the 49 countries and territories (77.6%) reported information, with data for circa 2014. (Eight of the countries reported information from an earlier year, but for simplicity and consistency, the wording “2014” is used throughout this article.) Among those 38, 28 of them (73.7%) reported the presence of human cases, with a total of 10 702 human leptospirosis cases recorded. The majority of the locations with zero cases present were Caribbean territories. Among the 21 countries and territories of the non-Latin Caribbean, 14 answered the request for information, with 8 of them reporting the presence of leptospirosis. Some of these countries and territories of the non-Latin Caribbean presented high rates of leptospirosis, such as with Trinidad and Tobago (363 cases and a rate of 27.0 per 100 000 population), Saint Vincent and the Grenadines (17 cases and a rate of 16.5 per 100 000 population), and Dominica (10 cases and a rate of 13.7 per 100 000 population).

### Overview of Latin America in 2014

**FIGURE 1.**
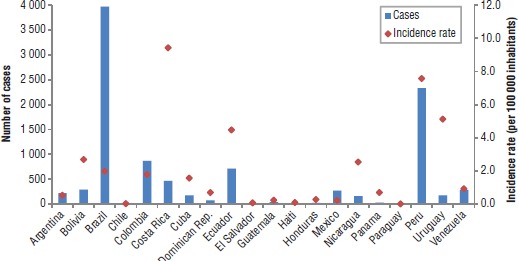
Number of cases of leptospirosis and incidence rate (per 100 000 inhabitants) in 20 countries of the Americas, 2014

***Source:*** Figure prepared by authors, using data from the PAHO Regional Core Health Data Initiative, based on country information.

In Latin America, 19 of the 21 countries and 2 out of the 4 territories reported information regarding leptospirosis to the PAHO Regional Core Health Data Initiative; one additional Latin American country submitted information later. Ultimately, a total of 20 countries and no territories from Latin America were included in our analysis, reporting a total of 10 088 human cases of leptospirosis, over a population of approximately 610.9 million (16) in 2014 (Figure 1). While Brazil had 40.2% of the reported cases, this country has almost half of the total population of Latin America. Brazil was followed by Peru (23.6%), Colombia (8.8%), and Ecuador (7.2%).

**FIGURE 2.**
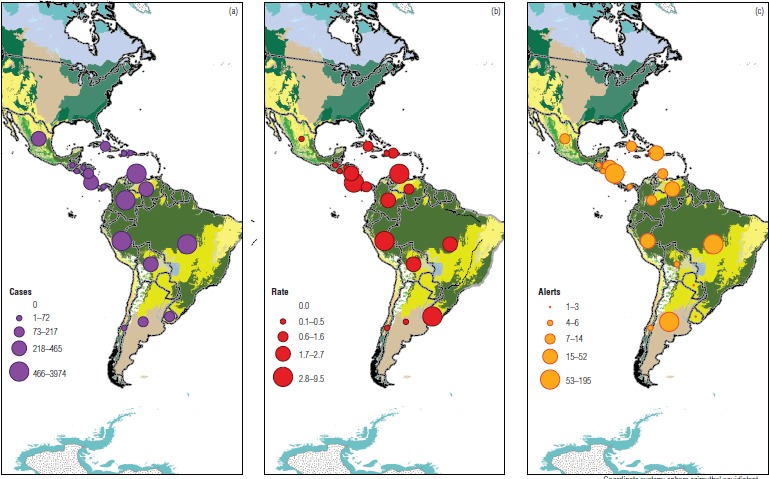
Human leptospirosis cases in 2014 (a), cumulative incidence rate (per 100 000 population) in 2014 (b), and leptospirosis alerts during 2010-2014 (c) in Latin America, presented on an ecoregions background

***Source:*** Maps developed by authors, based on the following information sources: human cases: PAHO, based on country information; cumulative incidence rates: calculated by authors; alerts: HealthMap; terrestrial ecoregions: Food and Agriculture Organization of the United Nations (FAO).

The cumulative incidence rate for Latin America in 2014 was estimated at 2.0 per 100 000 population, with a median of 1.6 (range, 0.02 to 9.42 per 100 000 population). The highest rates in 2014 were in Costa Rica, Peru, and Ecuador (Figure 2). The number of alerts from HealthMap for 2010 through 2014 was also incorporated into the analysis and compared with the 2014 surveillance data (Figure 2). Based on our exploratory analysis, comparing one year of surveillance data with a set of five years of alerts, we found that in general the indicators were similar. However, some countries with a high number of alerts did not have a high incidence rate.

Examining the incidence rates overlaid on the ecoregions map suggests that countries with large proportions of their territory composed of tropical and subtropical ecosystems are more likely to report the presence of leptospirosis. This pattern is well documented in the literature, given the epidemiology of the disease. However, some countries more distant from the equator, such as Uruguay, also presented high incidence rates.

Table 1 includes data on leptospirosis cases by sex in 15 countries that had 10 or more cases in 2014 and that also reported disaggregated information. On average, 65.1% of cases were males in these countries, with a median of 66.7% and a range of 40.5% to 90.9%.

In Table 1, the countries are ordered based on the percentage of female leptospirosis cases, from highest to lowest.Darker color palettes were used to illustrate *higher* quartiles of percentage of female leptospirosis cases, percentage of gross domestic product (GDP) from agriculture, and percentage of female employment in agriculture. In addition, darker color palettes were used to illustrate *lower* quartiles of GDP per capita (current US$), percentage of female literacy, and percentage of urban population.From our exploratory descriptive analysis for the purpose of generating questions for future studies, it appeared that countries with a higher percentage of female cases were more likely to have a lower GDP per capita, a higher percentage of GDP from agriculture, a higher percentage of female employment in agriculture, a lower percentage of female literacy, and a lower percentage of urban population. These hypotheses need to be tested further, using more comprehensive studies.

### Leptospirosis in Brazil, 2010-2014

Leptospirosis is endemic across Brazil throughout the year. From 2010 to 2014, an average of 4 176 human cases was reported annually by the Brazilian Ministry of Health, with a case fatality rate of 8.5%. A higher number of cases was reported in the southern states and in some northern states, especially from January to April, when precipitation is higher (Supplementary File 1) (17). A cumulative incidence rate of 2.1 per 100 000 population was observed in the country (which has 202.0 million inhabitants), with the large majority of cases (84.3%) reported in urban areas. On average, 77.6% of the cases were males, with the states having a median of 79.2% and a range of 57.1% to 87.7%.

In Brazil, surveillance activities are carried out throughout the country. Investigations are first conducted at the local level (municipality), then forwarded to the state and lastly to the national level. Large outbreaks are mainly detected after floods, contributing to the increase in the number of cases throughout the year, as occurred in Santa Catarina in 2008 (997 cases) and 2011 (700 cases), Pernambuco in 2011 (375 cases), São Paulo in 2011 (980 cases), Rondônia in 2014 (190 cases), and Acre in 2014 (1 196 cases). Brazil’s strategy to tackle the disease involves pinpointing priority municipalities (based on several indicators of incidence and mortality), putting in place responsive professionals, and identifying reference hospitals for patient care.

The main activities carried out by the leptospirosis epidemiological surveillance team in Brazil include: (a) monitoring the occurrence of cases and outbreaks and determining their spatial and temporal distribution; (b) adopting preventive and control measures; (c) conducting training and capacity-building activities for surveillance, diagnosis, and treatment of leptospirosis, as well as control of urban rodents, in partnership with states and municipalities; (d) preparing educational materials for the general public and clinicians (manuals, guides, posters); and (e) establishing partnerships and collaborations with research institutions to find new methods to fight the disease.

In order to reduce the number of false-positive laboratory confirmations, the Ministry of Health established the use of ELISA (IgM) in all state laboratories; serological testing using MAT in all regional laboratories; and the isolation of *Leptospira*, in combination with both testing methods, at the national laboratory. Death investigations are conducted using immunohistochemistry and/or polymerase chain reaction (PCR).

### Leptospirosis in Colombia, 2010–2014

In Colombia, various institutions collaborate on leptospirosis prevention and control. Among them are the Ministry of Health and Social Protection (which includes the National Health Institute) and the Ministry of the Environment (which includes the Colombian Environmental Institute). Leptospirosis is covered within the National Ten-Year Public Health Plan 2012–2021, which is a comprehensive strategy to manage and advance health promotion and disease prevention, including through control of vector-borne and zoonotic diseases.

Leptospirosis is a disease of compulsory notification in Colombia. Once a case has been detected at the local level, the primary care unit notifies the municipal health department, which then informs the district and state health departments, which are both responsible for reporting to the national level. Feedback is also distributed down the chain from the national to the local level, as well to health insurers. When necessary, national-level officials also inform agricultural authorities and international institutions.

**TABLE 1.** Cases of leptospirosis and selected indicators, by sex, for 15 countries in Latin America that had 10 or more cases in 2014 and that reported disaggregated information^a^

Country	Cases in males n (%)^b^ (mean and SD)	Cases in females n (%)^b^ (mean and SD)	GDP per capitac	Agriculture (% of GDP)^d^ (mean and SD)	% of female employment in agriculturee	% female literatef	% urban pop.^g^ (mean and SD)
Peru	943 (40.5)	1 386 (59.5)	6 541.00	7.4	5.5	91.7	78.6
Ecuador	378 (53.1)	334 (46.9)	6 345.80	9.1	20.3	93.5	63.7
Bolivia	156 (53.6)	135 (46.4)	3 124.10	13.0	h	93.6	68.5
Guatemala	21 (58.3)	15 (41.7)	3 673.10	11.5	10.9	76.3	51.6
Honduras	13 (59.1)	9 (40.9)	2 434.80	13.8	10.3	88.6	54.7
Nicaragua	95 (60.9)	61 (39.1)	1 963.10	20.5	h	83.2	58.8
Venezuela	179 (63.2)	104 (36.8)	12 771.60	5.5	1.6	96.2	89.0
Panama	18 (66.7)	9 (33.3)	11 948.90	3.5	8.5	94.4	66.6
Colombia	615 (70.9)	252 (29.1)	7 903.90	6.3	6.6	94.8	74.6
Brazil	3 038 (76.4)	936 (23.6)	11 384.40	5.6	10.1	92.9	85.7
Costa Rica	368 (79.1)	97 (20.9)	10 415.40	5.6	3.4	97.8	63.7
Dominican Rep.	61 (84.7)	11 (15.3)	6 163.60	6.2	2.8	92.3	79.0
Argentina	187 (86.2)	30 (13.8)	12 509.50	8.3	0.2	98.1	91.8
Cuba	151 (86.3)	24 (13.7)	6 789.80	5.0	7.7	99.8	77.1
Uruguay	159 (90.9)	16 (9.1)	16 806.80	8.1	4.4	98.8	95.3

***Source:***Table prepared by authors, based on the following information sources:^b^: PAHO, based on country information;^c^,^d^,^e^: World Bank;^f^,^g^: PAHO Regional Core Health Data Initiative.

a Countries are ordered based on the percentage of female leptospirosis cases, from highest to lowest. Darker color palettes correspond to *higher* quartiles of female cases ( ), of GDP from agriculture ( ), and of female employment in agriculture ( ), as well as to *lower* quartiles of GDP per capita ( ), of female literacy ( ), and of urbanization ( ).

b Cases of leptospirosis in 2014.

c Gross domestic product per capita (current US$).

d Percentage of gross domestic product from agriculture.

e Percentage of females who are employed in agriculture.

f Percentage of females who are literate.

g Percentage of the population in urban areas.

h NA = not available.

The country has a population of around 48.9 million. Over the 2010–2014 period, the country had an average of 1 252 confirmed cases reported annually and a cumulative incidence rate of 2.6 per 100 000 population. In Colombia, leptospirosis outbreaks are most common during the fourth trimester of the year, which is usually when the greatest number of floodeventshappen(SupplementaryFile2). In its first subnational administrative level, Colombia has 35 departments. Five departments in the mountainous Andean region reported 66.0% of the leptospirosis cases. Of the total number of cases reported in the country, 60.0% were males. Most cases were present in the municipal capitals. About 55.0% of the cases corresponded to individuals whose health care was subsidized by the State, that is, persons with the lowest income.

The most important risk factors identified by the country were related to sanitation and contact with animals. According to a Ministry of Health report, high-risk activities (from which more than one could be selected) included: contact with dogs (47.0%), rodents in the home (44.0%), rodents in the workplace (37.0%), lack of basic sanitation and a clean water source (27.0%), and storage of solid waste close to the residence (32.0%). Diagnostic tests for human cases at the local level are based on IgM ELISA and subsequently confirmed by MAT.

### Leptospirosis in Cuba, 2010–2014

The Cuban health system is based on the “family medicine” system, which a free, accessible, community-based program. The basis of the system consists of family doctors and nurses, with the recent addition of persons responsible for vector control. The Ministry of Public Health sets the guidelines and oversees the family medicine system at the national level.

Cuba’s national leptospirosis prevention and control program was established in 1964, based on research carried out during a large outbreak among sugarcane workers in rural areas. The program has a large intersectoral component, including the Ministry of Public Health, the Veterinary Medicine Institute, and other social and political organizations. The objective of the programs is to control leptospirosis in animals and to prevent and control leptospirosis in humans. After a high number of human cases was reported in 1994, an emergency plan was developed that included the distribution of Cubanproduced vaccines to at-risk groups.

The main activities of the leptospirosis program are to: provide treatment to all suspected human cases; provide leptospirosis vaccine to anyone who has a permanent risk and prophylaxis to people with temporary risk; separate, treat, or cull animals with clinical symptoms; vaccinate animals of economic importance and domestic animals, depending on the request of the owner; train health staff; control outbreaks and promote changes in behavior; and conduct epidemiological surveillance.

Cuba has a population of approximately 11.3 million. Leptospirosis is endemic in the country and has the potential for outbreaks. Between 2010 and 2014, an average of 232 human cases was reported annually (Supplementary File 3). The cumulative incidence rate for this period was of 2.1 per 100 000 population. Of the cases, 88.3% of them were males, and most of them were in economically productive ages. The median proportion of male cases was 90.7%, with the range for the provinces being from 80.2% to 100.0%. The most cited sources of infection were contact with soil and with domestic animals.

### Leptospirosis in Honduras, 2010–2014

Honduras is a tropical country with a great number of risk factors that favor the transmission of leptospirosis, which is one of the nine neglected tropical diseases prioritized within the Ministry of Health’s strategic national plan. The departments of Choluteca and Francisco Morazán, which are located in the central and southern part of the country, have the highest number of reported cases; the lowest numbers of cases are reported in the Caribbean coastal areas of the country (Supplementary File 4). Honduras has a population of 8.3 million people. Over the 2000–2014 period, approximately 55.0% of the leptospirosis cases occurred in rural areas. There was an average of 73 human cases per year reported, with a case fatality rate of 3.0%. The cumulative incidence rate calculated for 2000–2014 was 0.9 per 100 000 population.

Honduras reports leptospirosis cases throughout the year. However, higher numbers of cases are reported during the fourth quarter of the year, when the amount of rainfall and the number of floods increase in high-risk areas (18). During this same time of the year, surveillance activities are enhanced. Currently, improvements are being made to the differential diagnostics in the country.

All 20 health regions across the country perform leptospirosis surveillance, prevention, and control activities and notify the Zoonosis Unit. The objectives of the Ministry of Health, through the national leptospirosis program, are to:(a) promote the use of information for decision-making; (b) analyze the epidemiological information to characterizethe disease in the country; (c) monitor the serogroups and serovars circulating in the human and animal population; and (d) provide recommendations based on epidemiological surveillance and reorient the prevention and control measures.

Surveillance is based on identifying human cases (morbidity and mortality) and using laboratory testing of samples from wild and domestic animals to study possible sources of infection. A total of 1 625 health centers are part of the surveillance network, and 4 hospitals carry out sentinel surveillance. Leptospirosis cases are reported from the local level to the municipal, regional, and central levels. In addition, a weekly bulletin is available to the public. Laboratory surveillance is carried out through: (1) clinical samples from hospitalized patients; (2) blood samples from established risk groups to identify antibodies; (3) random blood samples from the general public to detect antibodies and to find the seroprevalence level in the population; (4) animal samples; and (5) environmental samples from water and soil. Serum is tested using ELISA IgM, rapid IgM/IgG tests, and MAT, while tissue and water samples are tested using culture. After laboratory results are obtained, a committee that also certifies cases of dengue is responsible for confirming or discarding suspected leptospirosis cases.

## DISCUSSION

Given that 2015 was the first year that data on human leptospirosis cases in the Region of the Americas were formally reported to the PAHO Regional Core Health Data Initiative, we were only able to conduct a preliminary analysis. However, this study demonstrates the importance of having surveillance systems and also how surveillance information can be used for evidence-based decision-making. It is important to note that the data completeness and the accuracy of the numbers reported depend on the performance of each country’s surveillance system, which differs widely among the countries of the Americas. In addition, usually only severe cases are laboratory confirmed and reported in the surveillance system. According to Costa and colleagues (1), severe leptospirosis cases account for 5% to 15% of all clinical infections.

For this reason, the focus of this analysis was not on the number of cases, but rather on illustrating the country’s capacity to detect and report cases of leptospirosis. The official, documented presence of leptospirosis in the majority of the countries and territories reporting highlights the importance of the disease as a public health problem for the Americas. It is imperative to start documenting the baselines of the disease in the Americas, as well as to strengthen laboratory capacity and, in the future, to standardize the case definition among countries.

According to a recent study of the global morbidity of leptospirosis (1), we would expect Latin America to have about 77 000 human cases per year, given that only around 10% of leptospirosis cases develop severe symptoms and a portion of them are probably misdiagnosed as other diseases, such as dengue (19), which has a high prevalence in Latin America. The total of approximately 10 000 cases reported to PAHO in this first year suggests that many countries in Latin America are moving in the right direction in terms of establishing strong surveillance systems and programs for this disease. The other countries, which reported a small number of cases, need to evaluate their clinical and laboratory diagnostic capacity for detecting cases, as well as their reporting systems.

For this study, we compared data from country surveillance systems with other types of information available—in this case, alerts from HealthMap. Combining data from a country’s surveillance system with other types of online, openaccess, and real-time information might be useful for defining risk areas.

Comparing these types of information raises other questions that need further research among individuals. For example, in countries with a considerable number of cases and very few leptospirosis alerts, could the disease be related more to occupation? Are endemic occupational cases more neglected and less noticeable than cases related to flood outbreaks? It is known that the risk of acquiring leptospirosis is higher in males than in females (1). However, in our preliminary analysis of leptospirosis cases disaggregated by sex, even though the average percentage of leptospirosis cases in the countries was higher among males, there was still an intriguingly wide range in the percentage of female cases, from 9.1% to 59.5%. By comparing this information on the gender distribution of leptospirosis with socioeconomic indicators for the disease, it may be possible to generate research questions for future studies. For example, could the percentage of female cases be related to the country’s economy and to the level of women’s participation in agriculture? Do leptospirosis cases in females increase in line with certain socioeconomic indicators?

Future studies will benefit from including other types of data to support this preliminary overview of the scenario of leptospirosis in Latin America. The use of “big data,” that is, putting together several sources and types of information, might lead to a better risk analysis with infectious diseases that have a strong environmental component.

It is important to note that during the 2010-2014 study period for the four highlighted countries, 2011 was the year with the highest number of leptospirosis cases reported in Brazil, Colombia, Cuba, and Honduras. During that year, La Niña (the El Niño–Southern Oscillation cold phase) contributed to extreme weather events around the globe (20), and total precipitation amounts were extremely high in northern South America (21). In Colombia, La Niña weather events affected millions of people, causing flooding and destruction of infrastructure (22). A study conducted in New Caledonia, a French overseas territory in the southwest Pacific Ocean, demonstrated a strong association between the El Niño–Southern Oscillation phenomenon and leptospirosis outbreaks (23). This type of analysis is very important, and it should be applied in future studies in Latin America.

The information we presented from the four selected countries that was disaggregated by the first subnational administrative level demonstrated that there has been progress in the countries’ capacity to detect cases, report data through their surveillance systems, analyze the information, and operate programs and control activities. This type of information from the Americas might be of interest to other regions of the world.

More studies about the epidemiological situation of leptospirosis in animals are needed in Latin America. The One Health approach, which integrates the human-animal-environment interface, is a perfect framework to better understand and fight this disease (12, 24, 25). This approach includes a collaborative effort across multiple disciplines, working locally, nationally, and globally to achieve optimal health for people, animals, and the environment (26—28). Proximity to some animals, such as dogs (29), increases the risk for leptospirosis. One study found that leptospirosis titers were present in 39.0% of cattle in the Brazilian state of Rio Grande do Sul (30).

There is an overall dearth of epidemiological data collected on neglected tropical diseases through active surveillance systems, as well as estimates of those diseases’ economic impact (31). The effort to include leptospirosis in the PAHO Regional Core Health Data Initiative is a step toward alleviating this lack of information. The countries and territories of the Americas need to continue to strengthen their surveillance systems for this disease. Nations and researchers need to work together to demonstrate the importance of this disease worldwide and to make leptospirosis more visible.

## Acknowledgments

This publication would not have been possible without the hard work of the country authorities and technical personnel at all levels who are involved in the fight against leptospirosis in the Americas. The authors would also like to thank the country representatives who participated in the International Workshop of the Oswaldo Cruz Institute/FIOCRUZ for Leptospirosis Research Based on Country Needs & 5th Global Leptospirosis Environmental Action Network (GLEAN) Meeting. In addition, appreciation goes to GLEAN members, PAHO colleagues in the country offices, and the PAHO Health Analysis Unit, especially Dr. Gerardo De Cosio. We also acknowledge Dr. Clovis Tigre, who dedicated his career to strengthening country surveillance systems and epidemiological efforts in the Region of the Americas.

## Disclaimer

Authors hold sole responsibility for the views expressed in the manuscript, which may not necessarily reflect the opinion or policy of the *RPSP/PAJPH* or PAHO.
